# Dorsolateral Prefrontal Cortex Activity during a Brain Training Game Predicts Cognitive Improvements after Four Weeks’ Brain Training Game Intervention: Evidence from a Randomized Controlled Trial †

**DOI:** 10.3390/brainsci10080560

**Published:** 2020-08-15

**Authors:** Rui Nouchi, Natasha Yuriko dos Santos Kawata, Toshiki Saito, Robin Maximilian Himmelmeier, Ryo Nakamura, Haruka Nouchi, Ryuta Kawashima

**Affiliations:** 1 Department of Cognitive Health Science, Institute of Development, Aging and Cancer (IDAC), Tohoku University, Sendai 980-8575, Japan; haruka.nouchi.e8@tohoku.ac.jp; 2 Smart Aging Research Center (S.A.R.C.), Tohoku University, Seiryo-machi 4-1, Sendai 980-8575, Japan; ryuta@tohoku.ac.jp; 3 Department of Functional Brain Imaging, Institute of Development, Aging and Cancer (IDAC), Tohoku University, Sendai 980-8575, Japan; natashakawata@med.tohoku.ac.jp (N.Y.d.S.K.); Toshiki.saito@med.tohoku.ac.jp (T.S.); robin.himmelmeier@stud.uni-goettingen.de (R.M.H.); nakamura.ryo.46@rtri.or.jp (R.N.); 4 University Medical Center Göttingen (UMG), Georg-August-University of Göttingen, Robert-Koch-Straße 40, 37075 Göttingen, Germany; 5 Safety Psychology, Human Science Division, Railway Technical Research Institute, Kokubunji 185-8540, Japan

**Keywords:** brain training, prediction, NIRS, cognitive improvement, randomized control trial, dorsolateral prefrontal cortex

## Abstract

Background: Recent studies have demonstrated that brain activities using NIRS (near-infrared spectroscopy) at baseline during cognitive tasks (e.g., N-back task) can predict the cognitive benefits of a cognitive training. In this study, we investigated whether brain activities during brain training game (BT) at baseline would predict benefits to cognitive functions after the intervention period. Methods: In a four-week double-blinded randomized control trial (RCT) 72 young adults were randomly assigned to one of the two groups: participants in the BT group played specific game, called the Brain Age. Participants in an active control group (ACT) played the puzzle game Tetris. We measured brain activity during the training games using two channel NIRS before the intervention period. Cognitive functions were tested before and after the four-week intervention period. Results: The BT showed significant improvements in inhibition, processing speed, and working memory performance compared to ACT. The left and right DLPFC (dorsolateral prefrontal cortex) brain activities during the BT at baseline were associated with improvements in inhibition and processing speed. Discussion: This randomized control trial first provides scientific evidence that DLPFC activities during BT at baseline can predict cognitive improvements after a four-week intervention period.

## 1. Introduction

Cognitive training aims to improve cognitive functions using cognitive tasks and games in life. Several cognitive training methods for young adults have been reported, such as working memory training [1], processing speed training [2], and a braining training game such as Brain Age [3]. A recent meta-analysis [4] and second-order-meta-analysis [5] have reported small positive effects on specific cognitive functions after cognitive training if mental processes are similar between the training task and a cognitive functions measure (near transfer). For example, working memory training has positive effects on working memory performance. Although the benefits of cognitive training remain controversial, cognitive training/brain training is attracting a great deal of attention.

Researchers in the cognitive training field are also interested in what factors can predict the benefits of cognitive training on cognitive functions. Recent neuroimaging studies focused on individual differences in brain activities [6,7]. The previous studies used brain activities at baseline to predict improvements in cognitive functions [6,7]. For example, one previous study reported that brain activities at baseline during the N-back task, which were measured by NIRS (near-infrared spectroscopy), were positively correlated with improved N-bask task performances in older adults with and without MCI (mild cognitive impairment) [8].

Previous studies have reported that cognitive benefits after cognitive training were predicted by brain activities. However, several issues remain unresolved. First, previous studies used brain activities during working memory tasks such as the N-back task. Brain activities during the N-Back task were correlated with an improvement in cognitive functions [6,7]. However, previous studies have focused on brain activities during cognitive assessments and it remains unknown, therefore, whether brain activities during cognitive training tasks at baseline would predict cognitive improvements. Second, participants in previous studies, which investigated whether brain activities can predict the benefits of cognitive training, comprised healthy children, older adults, or older adults with mild cognitive impairment [6,8]. Cognitive training has beneficial effects on children, as well as on young and older adults. Young adults often receive more beneficial effects after cognitive training [9]. However, it remains unclear whether brain activities can predict cognitive benefits in young adults. Third, previous studies did not conduct randomized control trials (RCTs), which are one of the gold standards for evaluating the effect of an intervention. Furthermore, previous studies did not use an active control group to control a new experience from using a new device, or social interaction with others during the intervention [6,8]. An RCT study using an active control group needs to be conducted to reduce these possible effects on the training benefits. 

For the first issues, we measured brain activities during cognitive training in young adults by NIRS. We used cognitive training with the brain training game Brain Age (developed by Nintendo) for two principal reasons. First, previous studies indicated the key brain region activated while playing Brain Age. Brain Age contains mathematical calculations and reading processes [10] and previous neuroimaging studies demonstrated greater brain activation at the dorsolateral prefrontal cortex (DLPFC) during mathematical calculations [11] and reading aloud [12]. Therefore, we assumed that the DLPFC would be activated while playing Brain Age. The second reason was that the training tasks in Brain Age do not directly use cognitive assessment tasks such as the N-back task. For example, working memory training typically uses working memory span tasks (e.g., N-back or digit span tasks) as the training task [6,8]. These studies used similar working memory span tasks to measure working memory performance before and after the training period. If we measured brain activities during the training task that were similar to working memory span tasks, it would be difficult to separate the brain activities that represent cognitive training performance and the brain activities that represent working memory span performance. Therefore, Brain Age would be suitable for measuring brain activities during cognitive training. Regarding the second issue, previous studies demonstrated that Brain Age can improve working memory, processing speed, and inhibition performance in young and older adults after a four-week intervention [3,10]. On that basis, we anticipated that Brain Age would lead to improvements in several cognitive functions in young adults. Regarding the third issue, we set an active control group that was asked to play a video game using the same device as the cognitive training game group. We chose Tetris due to its popularity and because it works on the same portable game device. Previous studies also used Tetris as the active control group in young and older adults [3,10]. In addition, a previous study demonstrated that participants in the Tetris group felt equal enjoyment and motivation as those in the Brain Age group [3]. 

The main purpose of this study was to investigate whether brain activities during brain training at baseline can predict the benefits of cognitive training in healthy young adults. Based on the previous evidence, we made the following hypotheses. First, the DLPFC would show greater activities while playing the brain training game compared to the puzzle game. Second, the brain training game would engender improvements in working memory, processing speed, and inhibition performance compared to the puzzle game [3,10]. Third, brain activities while playing the brain training game at baseline would predict these cognitive benefits in young adults.

## 2. Materials and Methods

### 2.1. Randomized Control Trial Design and Setting of This Trial

This RCT was conducted from November 2017 to May 2018 in Sendai, Japan. The study protocol was approved by the Ethics Committee of Tohoku University Graduate School of Medicine. This RCT was registered at the University Hospital Medical Information Network (UMIN) Clinical Trial Registry (UMIN000030499). All participants provided informed consent.

**Figure 1:**
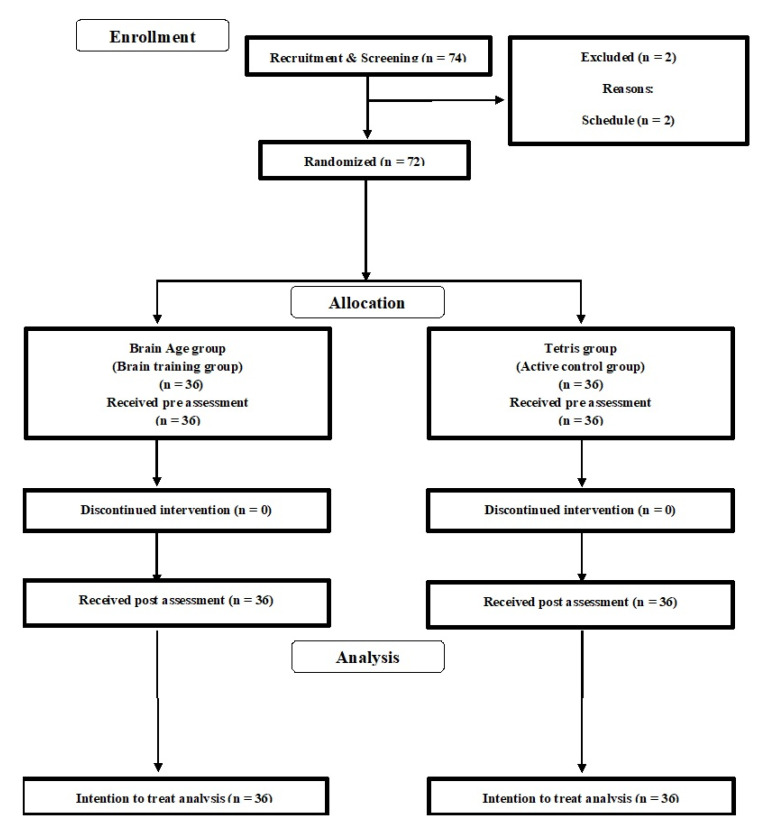
The Consolidated Standards of Reporting Trials (CONSORT) diagram.

We conducted a double-blinded RCT with an active control group. All participants and testers were blinded to the study hypothesis and the group membership of participants. The Consolidated Standards of Reporting Trials (CONSORT) statement (http://www.consort-statement.org/home/, see Supplementary Table S1) was used to report the study structure. The RCT design is presented in Figure 1.

### 2.2. Participants

We recruited undergraduate and graduate students using an online advertisement in the university. The inclusion and exclusion criteria were written on the flyers. A total of 74 interested participants contacted the research group by email (Figure 1) and participated in an orientation meeting. Two participants were excluded due to their schedule. During the meeting, one researcher (R.N.) explained the study details and all participants provided informed consent. The researcher then checked whether interested participants were eligible to participate in the study. No one was excluded at this stage. Then, the 72 participants were randomly assigned to either the brain training game (BT) or active control game (ACT) groups. Based on the intention to treat analysis (ITT), we did not recruit another participant. Table 1 presents the baseline characteristics of all participants (n = 72; 48 males, 24 females; average age = 21.63 years (SD = 1.26)). There was no significant difference in the baseline data between the two groups (two-sample *t*-test).

### 2.3. Inclusion and Exclusion Criteria

Based on our previous study [3], we used the following inclusion criteria: (1) right-handed; (2) native Japanese speakers; (3) 20–30 years of age; (4) not concerned about their memory functions and not using medications known to interfere with cognitive functions (including benzodiazepines, antidepressants, or other central nervous agents); (5) no history of diseases known to affect the central nervous system, including thyroid disease, multiple sclerosis, Parkinson’s disease, stroke, diabetes, and severe hypertension (systolic blood pressure over 180 mmHg, diastolic blood pressure over 110 mmHg); and (6) non-gamers who reported playing video games less than one hour a week over the previous two years. Participants who had participated in other cognition-related intervention studies were also excluded.

### 2.4. Sample Size

We calculated the sample size using the G*Power [13] applying the following procedure. We planned two main analyses. The first analysis was an ANCOVA, which checks whether the brain training game can improve cognitive functions compared to an active control game. A second analysis was a multiple regression analysis that investigated whether brain game-related brain activities can predict cognitive benefits. The primary analysis in this study was the multiple regression analysis. The ANCOVA analysis was a presupposition of the primary analysis.

The sample size was based on the multiple regression analysis because the primary purpose in this RCT was to investigate whether brain activities at baseline can predict cognitive benefits. Previous studies [8] reported that brain activities during working memory tasks such as N-back tasks were associated with cognitive training gains in healthy older adult and MCI patients. It reported the effect size from medium (f^2^ = 0.16) to large (f^2^ = 0.33). The population and tasks during brain measurements differed between the previous study and this study. Therefore, we expected a medium effect size (f^2^ = 0.15). We set α = 0.025 because we used the brain activities in the right and left channels separately to reduce the possibility of multicollinearity. To calculate the sample size, we used a linear multiple regression model using a two-tailed test, α = 0.025, and 0.80 power. The changes in cognitive functions were dependent values; the brain activities during brain training at baseline was the independent value, and sex and age were covariates. We assumed a dropout rate of approximately 0.05 based on our previous study [3]. The estimated sample size was 72 (*n* = 36 in each group).

### 2.5. Randomization

We randomly assigned the participants to the BT and ACT groups using an online randomization program (http://www.graphpad.com/quickcalcs/index.cfm). We stratified participants based on sex because there are sex differences in cognitive function [14] as secondary outcomes. We used blocked randomization (block size: 4) with an allocation ratio of 1:1.

### 2.6. Overview of the Intervention

In this study, we used a puzzle game for the ACT group. Therefore, participants in both groups had the same training period and a similar training setting, which reduced the effect of new experiences such as performing cognitive tasks on a new device and the effects of monitoring or maintaining the training schedule. 

The BT and ACT training games were played at home on a portable video game console (Nintendo 2DS LL) provided by the researchers with a video game soft (Brain Age for BT and Tetris DS for ACT). Before the intervention period, participants received instructions on how to use the console and play the training game. All participants played the training game using their console. The training duration time was recorded by the console. Participants were asked to play the training games alone for approximately 20 min every day for four weeks. After finishing each training game, participants were asked to write down all the game scores in a training note. At the end of the training period, participants reported their subjective feelings of satisfaction and enjoyment with the training game on a five-point Likert scale: 1 = strongly disagree; 2 = disagree; 3 = neither agree nor disagree; 4 = agree; 5 = strongly agree [15]. We measured brain activity while playing the video game using NIRS before the intervention period. Cognitive tests were conducted before and after the four-week intervention period. The console with the training game soft was returned on the assessment day after the completion of the intervention period.

### 2.7. Brain Training Group

We used Brain Age published by Nintendo as the BT game. In this study, we used eight brain training games. “(1) In Calculation × 20, participants are required to answer a total of 20 simple arithmetic calculations as quickly as possible. The questions include addition, subtraction, and multiplication. (2) In Calculation × 100, participants are required to answer a total of 100 questions as quickly as possible. The questions include addition, subtraction, and multiplication. (3) In Reading Aloud, participants are required to read excerpts from Japanese classical literature aloud. (4) In Syllable Count, some sentences written in a combination of kanji and kana are presented. Participants are required to count the total number of kana letters after translating from kanji to kana. (5) In Low to High, numbers in boxes are first presented for several seconds. Then, participants are required to select the boxes from the lowest to the highest number. (6) In Head Count, participants watch scenes in which people enter or leave a house. Participants are then required to answer how many people are in the house at the end. (7) In Triangle Math, three numbers are presented on a top-line (e.g., 5, 7, 2), two mathematical operations on a second line (e.g., +, +), and one mathematical operation (e.g., +) on the last line. Firstly, participants are required to solve the first formula (5 + 7) using the first two numbers (5, 7) in the first line and the first mathematical operation (+) in the second line, and then the second formula (7 + 2) using the last two numbers (7, 2) in the first line and the last mathematical operation (+) in the second line. Participants are then required to solve the last formula using the answer to the first formula (12), the answer to the last formula (9), and the mathematical operation (+) in the last line. In this case, participants give the final answer (21). (8) In Time Lapse, two analog clocks are presented. Participants are then required to calculate the difference in time between the two clocks. At the beginning of the game, participants can only perform three pieces of training (Calculation × 20, Calculation × 100, and Reading Aloud)” [3,10]. New training games are added to the game list after training for several days. After playing the games, participants were asked to write down their game performances on each training game in a training book.

### 2.8. Active Control Training Group

We used Tetris as the ACT game, based on previous studies [3,10]. Tetris is a popular block-puzzle game in which blocks drop from the top of the screen and participants can rotate and move the block, fitting them together to make a complete line. If the line is completed with no gap, participants acquire game points. Tetris aims to acquire high game scores by forming complete lines. The active control group was designed to control for using a new device, playing a video game, and keeping an intervention schedule.

### 2.9. fNIRS Measurements

Prefrontal brain activities while playing the video game were measured using HOT-1000 (NeU, Japan: https://neu-brains.co.jp/service/equipments/hot-1000/). The NIRS device can measure prefrontal brain activities [16,17].

HOT-1000 uses only a single wavelength of 810 nm. The HOT-1000 has two dual source-detector (SD) optode sets, which consist of one light source and two light detectors. The two light detectors are placed at a distance of 1 cm and 3 cm from the light source. The signal of the 1 cm SD pair is called a shallow signal. It represents an auxiliary measurement signal from shallow tissues like scalp and skull and is not affected by blood flow changes to the brain. The signal of 3 cm SD pairs is called a deep signal, which represents the brain signal in the cortex. HOT-1000 measures the concentration change of total hemoglobin (total Hb) on each SD. We set the two SD optnodes at the left and right dorsolateral prefrontal cortex (DLPFC: BA46), with the 3 cm detector 3 cm left or right from the Fpz position in the 10–20 system [17]. 

We measured brain activities while playing the brain training or active control games. We used the following block design with resting (R) and playing video game (V) blocks: R-V-R-V-R. During the resting block, participants were shown a fixation on the screen and asked to rest. During the video game block, participants in the brain training group were asked to play Calculation × 100 in Brain Age for 30 s. Participants in the active control group were asked to play Tetris for 30 s. 

### 2.10. fNIRS Data Preprocessing

First, to reduce potential drift and physiological noises from the NIRS signals, those signals were detrended and low-pass filtered (cut-off at 0.1 Hz). Second, we used dual source-detector regression to calculate the neural signals [18]. In the dual source-detector regression, systemic and motion-related noises regressed out the shallow signal component provided by the 1-cm-SD channels from the deep signals from 3-cm-SD channels [16]. Then, the NIRS signals while playing video games were baseline-corrected by subtracting the average NIRS signals of the preceding rest periods (of the 30 s). Finally, we averaged the baseline-corrected NIRS signals in each group.

### 2.11. Cognitive Functions

We investigated the performance of processing speed, attention, inhibition, short-term memory, working memory, and episodic memory. It took approximately 1.5 h to complete all the cognitive tests.

To briefly check the participants’ reading ability and IQ, we used the JART [19], which is a Japanese version of the National Adult Reading Test (NART), consisting of 25 Kanji (Chinese characters) compound words. The reading stimuli were randomly printed for reading. Participants were asked to write the pronunciation of each Kanji compound word.

To assess processing speed performance, we used digit symbol coding (Cd) and symbol search (SS) from the WAIS-III [20]. The following descriptions of Cd and SS were reproduced from our earlier report [21]: “For Cd, the participants were shown a series of symbols that were paired with numbers. Using a key within a 120 s time limit, participants drew each symbol under its corresponding number. The primary measure of this test was the number of correct answers. In SS, participants visually scanned two groups of symbols (a target group and a search group) and indicated whether either of the target symbols matched any symbol in the search group. Participants responded to as many items as possible within a 120 s time limit. The primary measure of this test was the number of correct answers.”

To measure inhibition performance, we used a reverse Stroop task (rST) and Stroop task (ST) [22]: “In the reverse ST, in the leftmost of six columns, a word naming a color is printed in another color (for example, “red” is printed in blue letters); the other five columns are each filled with five different colors from which participants must check the column whose color matches the written word in the leftmost column. In the ST, in the leftmost of six columns, a word naming a color was printed in another color (e.g., “red” was printed in blue letters); the other five columns contain words naming colors. Participants were required to check the column containing the word naming the color of the word in the leftmost column. The primary measure for this task was the number of correct items” [21].

To measure attention performance, we conducted the digit cancellation task (D-CAT). The following descriptions of the D-CAT are reproduced from our earlier report [3]: “The test sheet consists of 12 rows of 50 digits. Each row contains five sets of numbers 0–9 arranged in random order. Thus, any one digit would appear five times in each row with randomly determined neighbors. The D-CAT consists of three such sheets. Participants were instructed to search for the target number that had been specified to them and to delete each one with a slash mark as quickly and as accurately as possible for 1 min until the experimenter sent a stop signal. The primary measure of this test was the number of hits.” 

To measure short-term memory performance, we used the digit span forward (DS-F) and digit span backward (DS-B) tasks. DS-F and DS-B are the subtests of the WAIS-III [20]. The following descriptions of the DS-F and DS-B are reproduced from our earlier report [21]: “For the DS-F, participants repeated numbers in the same order as they were read aloud by the examiner. For the DS-B, participants repeated numbers in the reverse order of that presented aloud by the examiner. In both tasks, the examiner read a series of number sequences which the participant was required to repeat in either forward or reverse order.” The primary measures of this test were the digit number length. The maximum digit number length in the DS-F was 8, and that of the DS-B was 7. 

To measure working memory performance, we used the Letter–Number Sequence (LNS) and the working memory updating task (WMU). LNS is a subtest of the WAIS-III [20]. The description of LNS is reproduced from a previous study [3]. For LNS, “the examiner read a combination of letters and numbers; then participants were asked to recall numbers first in ascending order, followed by the letters in Japanese alphabetical order. If participants responded with letters first, followed by numbers, but with all of them in the correct sequence, credit was awarded. LNS begins with the simplest level of a three-letter number sequence. There are five sets of letters and numbers in increasing length, and each set consists of three trials (total 15 trials). The maximum raw score is 15” [3]. In WMU, participants were asked to remember the last three digits of the list (3-digit condition) and the last four digits of the list (4-digit condition). The order of the condition was fixed, with the 3-digit condition coming first. For example, in the 3-digit condition, participants listened to the digit list (e.g., 9-6-3-4-2) and then wrote down the last three digits of the list (e.g., 3-4-2). The list length for the 3-digit condition ranged from three to nine digits. The list length for the 4-digit condition ranged from 4 to 10 digits. Each condition had eight lists (total 16 lists). The primary outcome measure was the total number of correct answers (Max = 16).

To measure episodic memory, we used the logical memory (LM) subtest of the WMS-R [23]: “LM consists of two short-paragraph-length stories (Story A and Story B). For the LM activity, participants were required to memorize one of the two stories. The stories were scored in terms of the number of story units recalled, as specified in the WMS-R scoring protocol. We used either Story A or Story B. The primary measure for this task was the number of correct story units recalled” [21]. We checked the performances of immediate recall and delayed recall memory.

### 2.12. Data Analyses

All participants were included based on the intention to treat (ITT) principle. First, we calculated the change scores in cognitive functions (post-intervention score minus pre-intervention score). In addition, we conducted preprocessing for fNIRS data. After that, we conducted three types of analysis.

To investigate group differences in brain activities while playing video games at the baseline, we separately performed ANCOVA with permutation tests for the brain activities at the right and left DLPFC. In the ANCOVA, the brain activities were the dependent variable, while the group was the independent variable, and age and sex were used as covariates. 

To check whether brain training leads to improvements in cognitive functioning, we performed ANCOVA with permutation tests for all change scores. In the ANCOVA, the change score was the dependent variable, the group was the independent variable, pre-scores were the dependent variable, and age and sex were used as covariates. We performed permutation tests because they are suitable for small sample analyses and are distributed freely. Previous studies used a similar method in RCT [3,15]. All ANCOVAs with the permutation test were performed using the “aovp” function in the lmPerm package (http://cran.r-project.org/web/packages/lmPerm/index.html). 

To investigate whether brain activities during brain training games can predict improvement to cognitive function, we used the k-fold cross-validation method (k = 5). Firstly, we separated the data in the BT group into training sets (80%) and test sets (20%). To make a prediction model, we separately performed a permutation multiple regression analysis for brain activities at the left and right DLPFC using the “lmp” function in the lmperm package in the training sets. In the prediction model, we used the left or right DLPFC as the dependent values, the change score in cognitive function as the independent value, while the pre-score in the dependent variable at baseline, age, and sex were used as covariates. Next, we calculated the RMSE (Root Mean Square Error) of the prediction modes using the brain activities at the left and right DLPFC in the test sets from the 5-fold cross-validation method. 

In these analyses, significance was inferred for *p* < 0.05 for multiple comparison method. We used a false discovery rate (FDR) correction method [24] to adjust all pooled p values using p.adjust in R. All analyses were performed using software (R ver. 3.52).

## 3. Results

There was no significant difference in the average number of training days between the BT (*M* = 25.2 days, *SD* = 0.75) and ACT (*M* = 25.5 days, *SD* = 0.65) groups. We evaluated participants’ satisfaction and enjoyment after the intervention using a 5-point scale. There was no significant difference in the average satisfaction scores between the BT (*M* = 3.91, *SD* =0.44) and ACT (*M* = 3.82, *SD* = 0.43) groups and in enjoyment scores between the BT (*M* = 3.76, SD =0.64) and ACT (*M* = 3.78, *SD* = 0.64) groups. There was no significant difference between the two groups for the measures at baseline (Table 1). There was no drop out during the intervention period. We analyzed all data in 72 participants (BT: *n* =36, ACT: *n* =36).

**Figure 2:**
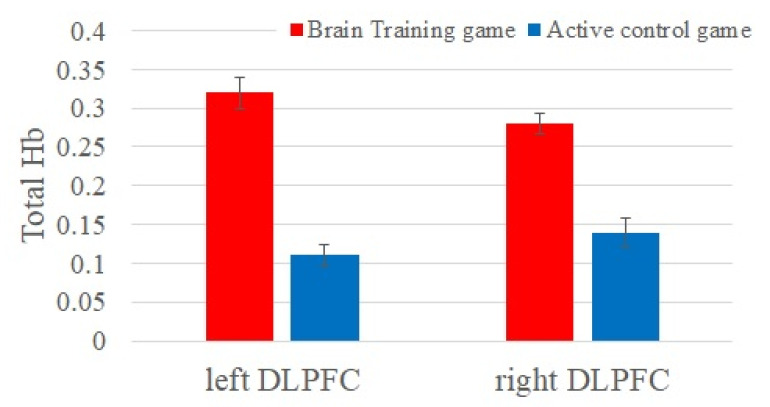
Brain activities measured by near-infrared spectroscopy (NIRS) while playing video games. Note: error bars are standard error (SE).

First, we investigated group differences in brain activities while playing video games at baseline (Figure 2). The BT group showed greater brain activities at the left and right DLPFC compared to the ACT (left DLPFC: *F*(1, 67) = 6.43, *η*^2^ = 0.06, *adjusted p* = 0.026 and right DLPFC: *F*(1, 67) = 5.77, *η^2^* = 0.02, *adjusted p* = 0.021).

Next, we checked the benefits of BT on cognitive functions (Table 2). For cognitive abilities, the BT group showed a significant improvement in processing speed performance (Cd: *F*(1, 67) = 5.94, *η^2^* = 0.05, *adjusted p* = 0.039 and SS: *F*(1, 67) = 5.54, *η*^2^ = 0.02, *adjusted p* = 0.039), in episodic memory (LM: *F*(1, 67) = 5.02, *η*^2^ = 0.04, *adjusted p* = 0.039 and LM delay: *F*(1, 67) = 16.45, *η*^2^ = 0.08, *adjusted p* = 0.000), in working memory (LNS: *F*(1, 67) = 19.67, *η*^2^ = 0.12, *adjusted p* = 0.000 and WMU: *F*(1, 67) = 3.58, *η*^2^ = 0.028, *adjusted p* = 0.039), and in inhibition (rST: *F*(1, 67) = 4.50, *η*^2^ = 0.05, *adjusted p* = 0.039 and ST: *F*(1, 67) = 2.93, *η*^2^ = 0.032, *adjusted p* = 0.043). There were no significant changes in attention or short-term memory domains. 

Finally, we investigated whether brain training-related activities predict improvements to cognitive functions. The results of the BT group showed that the brain activities in the left and right DLPFC at baseline were positively correlated with improvement of processing speed scores (Cd score (*standardized β* = 0.42, *t* = 2.54, *p* = 0.01) and SS score (*standardized β* = 0.413, *t* =2.78, *p* = 0.00). In addition, the BT group showed a significant positive correlation between brain activities in the left and right DLPFC and improvement of working memory performance (LNS score (*standardized β* = 0.36, *t* = 2.96, *p* = 0.01) and WMU score (*standardized β* = 0.439, *t* = 3.61, *p* = 0.00). There was no significant correlation between the improvement of episodic memory and DLPFC activities and inhibition performance and DLPFC activities in the BT group. Moreover, no significant result was found in the multiple regression analyses of the ACT group. The five-fold cross-validation showed that the prediction model using the left DLPF activities predicted the improvements to cognitive functions (RMSE = 0.75) compared to that using the right DLPFC activities (RMSE = 1.75).

## 4. Discussion

We investigated whether brain activities during the brain training game at baseline could predict improvement in cognitive functions after the four-week brain training period. There were three main findings. First, the BT group showed greater brain activities at the right and left DLPFC than the ACT group. Second, the BT group showed cognitive improvement in inhibition, processing speed, working memory, and episodic memory after the four-week intervention period. Third, the brain training-related brain activities at DLPFC were positively associated with cognitive improvement of working memory and processing speed after the intervention period. Taken together, the current results extend those of previous studies by demonstrating an association between brain activities at baseline and improvements to cognitive functions after the cognitive training. These findings are discussed below.

The first main finding is that playing the BT showed greater brain activities at the right and left DLPFC than playing the ACT. This result is consistent with previous neuroimaging studies using mathematical calculations [11] and Tetris [25]. During BT, participants were asked to solve simple calculations as quickly as possible. Previous neuroimaging studies using calculations showed greater activations in the left and right DLPFC [11]. In addition, participants in the ACT were asked to play the Tetris game, which requires mental rotation processes. One fMRI study [25] reported that playing Tetris activated the premotor area, the precuneus, and the superior parietal lobule, but not the DLPFC. However, this result first demonstrated that playing the BT showed greater DLPFC activation measured by NIRS compared to playing the other video game.

The second main finding is that the four-week BT showed significant improvement in inhibition, processing speed, working memory, and episodic memory in healthy young adults. These results are consistent with those of previous studies using the same BT (Brain Age) in healthy adults [3,10]. Previous studies repeatedly demonstrated that four weeks of BT using Brain Age improved the inhibition, processing speed, and working memory performance of healthy young and older adults [3,10]. In addition, our study first showed that Brain Age led to significant improvement in episodic memory performance in young adults. A previous study using a similar paper-pencil version reading aloud and simple calculation training reported that 20 weeks of cognitive training can improve episodic memory performance in healthy adults [26]. Previous studies using Brain Age did not measure episodic memory performance [3,10] and it is difficult to conclude that Brain Age has a positive effect on episodic memory. However, these results indicate that the short-term cognitive training with Brain Age has beneficial effects on a wide range of cognitive functions.

Improvements in processing speed, inhibition, working memory, and episodic memory are explained by the overlapping hypothesis [15,27,28], which assumes that cognitive functions will improve with cognitive training when training tasks and untrained measures (e.g., cognitive functional tests) share common mental and behavioral processes [27]. Based on the Cattel–Horn–Carroll (CHC) model [29], behavioral and mental processes can be divided into three levels: narrow, broad, and general abilities. The training tasks of BT are mental calculations and reading aloud. Calculation and reading are general abilities that include several narrow and broad abilities. Previous studies have demonstrated that calculation and reading performance are correlated with broad abilities, such as inhibition, working memory, and episodic memory [30,31]. In addition, we asked participants to complete the training games as quickly as possible. Based on the overlapping hypothesis, improvements in cognitive function performance are sufficient to explain the following: (1) the mathematical calculation and reading training in BT require mental processes including processing speed, inhibition, working memory, and episodic memory components. (2) By playing BT, these mental processes are expected to be facilitated and enhanced. (3) The BT and measurements of cognitive functions shared similar mental processes. Therefore, processing speed, inhibition, working memory, and episodic memory were directly improved because mathematical calculation and reading training required these cognitive processes.

The third main finding is that the left and right DLPFC activities while playing the BT were associated with improved processing speed and working memory performances. In addition, from the five-fold cross-validation method, the left DLPFC activities while playing the BT better predicted the improvement of processing speed and working memory performance than the right DLPFC activities. Previous studies have shown that brain activities during training tasks were associated with improvements in trained task performance [6,7]. For example, a previous study demonstrated that DLPFC activities during working memory training were positively associated with improvement to working memory performance [8]. However, this study expanded the previous results demonstrating that brain activities during a brain training game can predict several improvements in cognitive performance that were not directly trained. It should be noted that the brain activities while playing the BT did not predict all improvements in cognitive functions. 

These findings that the DLPFC activities during BT at baseline predicted cognitive improvements in working memory and processing speed can also be explained by the aforementioned overlapping hypothesis. The mental process of playing BT and improved cognitive functions shared similar neural and brain activities at the DLPFC. Our results demonstrate greater brain activities at the DLPFC while playing the BT. Previous neuroimaging studies have reported that DLPFC activations play an important role in working memory and processing speed [32,33]. Therefore, greater brain activities while playing BT at the DLPFC would be an indicator of cognitive plasticity after BT. Although previous studies did not measure the brain activities at baseline during the training task, they demonstrated that greater activities at the DLPFC during a working memory task were significantly associated with improved working memory performance [8]. Our study measured the brain activities at baseline during the training task itself (BT). The brain activities while playing BT were associated with improvements in both processing speed and working memory. This suggests that we will be able to predict the cognitive benefits of several cognitive functions using the brain activities of training tasks.

This study has several limitations. First, our participants comprised healthy young adults. Previous studies have demonstrated that BT has a positive effect on cognition in both healthy young and older adults. It is important to investigate whether brain activities while playing BT can predict improvements in cognitive function in healthy older adults. Second, we measured only brain activities at the DLPFC using the two-channel portable NIRS because our BT was mainly expected to activate the DLPFC. In addition, the portable two-channel NIRS allowed easy measurement of brain activities at the prefrontal cortex. However, it remains unclear whether other brain regions such as the parietal and temporal cortexes can predict improvement in cognitive functions after four-weeks cognitive training (BT). Further studies should investigate whether brain activities in the frontal, parietal, and temporal cortexes would predict improvements in cognitive function in healthy older adults. Third, it should consider lower effect sizes in the improvements in cognitive functions and the DLPFC activities at the baseline. The improvements in cognitive functions showed around medium effect size (from 0.04 to 0.10 in *η*^2^*)*. Our intervention period was shorter than previous cognitive training studies (e.g., 12 weeks). The shorter intervention period would be a possibility of the small effect size in this study. It would be important to investigate whether the intervention period would affect the benefits of BT. For the DLPFC activities at the baseline, the effect sizes were from small (*η*^2^ = 0.02) to medium (*η*^2^ = 0.06). We used the block design to measure brain activities by the NIRS. We used two blocks for playing the video game. The number of blocks would affect the S/N ratio in the NIRS signals. To increase the effect size of the DLPFC activities, it would be better to use more blocks for playing a video game.

## 5. Conclusions

In conclusion, we conducted an RCT to investigate whether brain activities during a brain training game can predict improvement in cognitive function. Our results replicated that BT led to improvements in inhibition, processing, working memory, and episodic memory. In addition, our results showed that the brain activities at the DLPF during BT were associated with improved processing speed and inhibition performance. From five-fold cross-validation, the prediction model using the left DLPFC activities was better than that using the right DLPFC activities. These results indicate that greater brain activities at baseline can predict the benefit of BT.

## Figures and Tables

**Table 1:** Demographic information and cognitive function scores of both groups at baseline.

	BT Group		ACT Group		Effect Size (*d*)	*p*-Value
	Mean	SD	Mean	SD		
Demographic Information						
Year	20.19	0.32	20.87	0.22	0.11	0.87
JART (Score)	21.22	1.4	20.19	1.7	0.16	0.77
Processing Speed						
Cd (Number)	61.48	10.36	58.07	10.70	1.05	0.24
SS (Number)	30.81	2.94	29.18	5.13	0.81	0.15
Executive Functions (Inhibition)						
rST (Number)	38.85	5.43	36.14	5.75	1.15	0.08
ST (Number)	27.41	7.63	23.93	8.02	1.24	0.11
Short-Term Memory						
DS-F (Digit Number)	5.30	1.03	5.18	1.12	0.11	0.69
DS-B (Digit Number)	4.04	1.13	3.89	0.83	0.15	0.59
Working Memory						
LNS (Number)	6.54	1.21	6.03	1.41	0.11	0.62
WMU (Number)	7.12	1.08	7.42	1.36	0.12	0.55
Attention						
D-CAT (Number)	4.04	1.13	3.89	0.83	0.15	0.59
Episodic Memory						
LM Immediate (Score)	8.74	4.13	9.07	3.53	0.17	0.75
LM Delay (Score)	8.26	3.90	8.54	3.71	0.14	0.79

Note: BT = Brain training game, ACT = active control game, SD = standard deviation, JART = Japanese version of the National Adult Reading Test, Cd = digit symbol coding, SS = symbol search, rST = reverse Stroop task, ST = Stroop task, DS-F = digit span forward, DS-B = digit span backward, LNS = letter number sequence, WMU = working memory updating task, D-CAT = digit cancellation task, LM = logical memory.

**Table 2:** Change scores of cognitive function of both groups.

	BT Group		ACT Group		Effect Size *(eta^2^)*	Adjusted *p*-Value	Non-Adjusted *p*-Value
	Mean	SD	Mean	SD			
Processing Speed							
Cd (number)	4.48	4.64	0.68	5.56	0.10	0.039	0.010
SS (number)	3.15	3.16	0.79	3.67	0.09	0.039	0.007
Executive Functions (Inhibition)							
rST (Number)	4.00	4.88	0.89	5.01	0.06	0.039	0.015
ST (Number)	2.70	3.23	0.29	4.48	0.06	0.042	0.012
Short-term Memory							
DS-F (Digit Number)	0.00	1.39	0.29	1.01	0.02	0.273	0.156
DS-B (Digit Number)	0.33	0.96	0.21	0.99	0.00	0.323	0.369
Working Memory							
LNS (Number)	3.21	1.76	0.78	1.98	0.05	<0.001	0.013
WMU (Number)	2.17	0.87	0.66	0.95	0.04	0.039	0.011
Attention							
D-CAT (Number)	12.37	25.13	11.14	17.79	0.00	0.314	0.381
Episodic Memory							
LM Immediate (Score)	3.21	3.32	−0.18	2.84	0.01	0.039	0.011
LM Delay (Score)	4.31	3.06	−0.32	3.32	0.01	<0.001	0.014

Note: BT = Brain training game, ACT = active control game, SD = standard deviation, JART = Japanese version of the National Adult Reading Test, Cd = digit symbol coding, SS = symbol search, rST = reverse Stroop task, ST = Stroop task, DS-F = digit span forward, DS-B = digit span backward, LNS = letter number sequence, WMU = working memory updating task, D-CAT = digit cancellation task, LM = logical memory.

## Supplementary Materials

The following are available online at https://www.mdpi.com/2076-3425/10/8/560/s1, Table S1: Details of CONSORT checklist of this study.
